# Structural basis for raccoon dog receptor recognition by SARS-CoV-2

**DOI:** 10.1371/journal.ppat.1012204

**Published:** 2024-05-06

**Authors:** Fu-Chun Hsueh, Ke Shi, Alise Mendoza, Fan Bu, Wei Zhang, Hideki Aihara, Fang Li

**Affiliations:** 1 Department of Pharmacology, University of Minnesota Medical School, Minneapolis, Minnesota, United States of America; 2 Center for Emerging Viruses, University of Minnesota, Minneapolis, Minnesota, United States of America; 3 Department of Biochemistry, Molecular Biology and Biophysics, University of Minnesota, Minneapolis, Minnesota, United States of America; University of Utah, UNITED STATES

## Abstract

Since the COVID-19 outbreak, raccoon dogs have been suggested as a potential intermediary in transmitting SARS-CoV-2 to humans. To understand their role in the COVID-19 pandemic and the species barrier for SARS-CoV-2 transmission to humans, we analyzed how their ACE2 protein interacts with SARS-CoV-2 spike protein. Biochemical data showed that raccoon dog ACE2 is an effective receptor for SARS-CoV-2 spike protein, though not as effective as human ACE2. Structural comparisons highlighted differences in the virus-binding residues of raccoon dog ACE2 compared to human ACE2 (L24Q, Y34H, E38D, T82M, R353K), explaining their varied effectiveness as receptors for SARS-CoV-2. These variations contribute to the species barrier that exists between raccoon dogs and humans regarding SARS-CoV-2 transmission. Identifying these barriers can help assess the susceptibility of other mammals to SARS-CoV-2. Our research underscores the potential of raccoon dogs as SARS-CoV-2 carriers and identifies molecular barriers that affect the virus’s ability to jump between species.

## Author summary

Raccoon dogs are thought to have been a crucial link in the chain that led to the spread of the COVID-19 virus to humans. The key to a coronavirus moving from one species to another is its spike protein’s ability to bind to receptors on the cells of the new host. In our research, we investigated the interaction between the spike protein of the COVID-19 virus and the ACE2 receptor in raccoon dogs, using both structural and biochemical techniques. We found that the ACE2 receptor in raccoon dogs does indeed work as an effective receptor for the virus, but there are critical differences when compared to the human ACE2 receptor, which may act as barriers to the virus making the jump between raccoon dogs and humans. To our knowledge, this study is the first in-depth examination of these cross-species transmission barriers for the COVID-19 virus, shedding light on how other mammals might be impacted by the virus. Our findings indicate that raccoon dogs could potentially carry and pass the COVID-19 virus on to humans, providing foundational knowledge for how the virus can navigate between different species.

## Introduction

Which animals were responsible for transmitting SARS-CoV-2 to humans, sparking the COVID-19 pandemic in late 2019? Several species are under scrutiny as potential culprits, including bats, pangolins, and raccoon dogs [1]. Raccoon dogs came under suspicion after their DNA was detected alongside SARS-CoV-2 genetic material in samples from the Huanan Seafood Market in Wuhan City [2], which is widely considered the pandemic’s point of origin [3]. Notably, raccoon dogs and palm civets were also implicated in the transmission of SARS-CoV-1, a virus related to SARS-CoV-2, to humans during the 2002–2003 SARS outbreak [4]. This study delves into the potential involvement of raccoon dogs in the emergence of COVID-19 by exploring the molecular and structural interactions that enable prototypic SARS-CoV-2 to bind to its raccoon dog’s ACE2 receptor.

Receptor recognition by coronaviruses is one of the most important determinants of their host range and tropism [5–7]. The coronavirus spike protein mediates viral entry by binding to its host receptor and then fusing viral and host membranes. A defined receptor-binding domain (RBD) in the spike protein is responsible for receptor recognition. The RBDs of SARS-CoV-2 and SARS-CoV-1 recognize the same receptor protein, angiotensin-converting enzyme 2 (ACE2) [8–12]. We previously determined the crystal structures of these RBDs from prototypic strains each complexed with human ACE2, establishing the structural framework for understanding the coronavirus/receptor interactions [9,11]. Subsequently, we determined the structures of the RBDs from various strains of SARS-CoV-1 in complex with the ACE2 receptors from different host species, including palm civets [13,14]. We also determined the RBDs from different strains of SARS-CoV-2 in complex with the ACE2 receptors from different host species, such as mice [15–17]. Our previous structural studies collectively established RBD/ACE2 interactions as a critical interspecies barrier for coronaviruses to transmit from one host species to another [5–7]. Other groups have also determined the structures of SARS-CoV-2 RBD complexed with ACE2 from different host species including domestic dogs, minks, cats, palm civets, and horses [18–23]. These studies delineated the structural determinants that allow SARS-CoV-2 to infect these host species.

Because of the importance of receptor recognition to the host range of coronaviruses, structural insights into the binding interactions between SARS-CoV-2 and raccoon dog ACE2 are essential for evaluating the role of raccoon dogs in potentially transmitting the virus to humans. Although raccoon dogs have been shown to contract SARS-CoV-2 [24–26], the structural characterization of the interactions between SARS-CoV-2 RBD and raccoon dog ACE2 has yet to be described. Moreover, it is unclear how structural variations in the ACE2 receptors among various host species affect their ability to act as receptors for SARS-CoV-2, leaving a significant gap in our understanding of the molecular factors that create cross-species transmission barriers for SARS-CoV-2.

In this study, we determined the crystal structure of SARS-CoV-2 RBD complexed with raccoon dog ACE2 (both the RBD and ACE2 had been engineered to facilitate crystallization) and also characterized the biochemical interactions between SARS-CoV-2 spike and raccoon dog ACE2. We further identified the structural variations between raccoon dog ACE2 and human ACE2 that account for their different activities as receptors for SARS-CoV-2. This research sheds light on the possibility that raccoon dogs could transmit SARS-CoV-2 to humans and delineates the interspecies barriers for SARS-CoV-2 to transmit between the two species.

## Results

**Fig 1:**
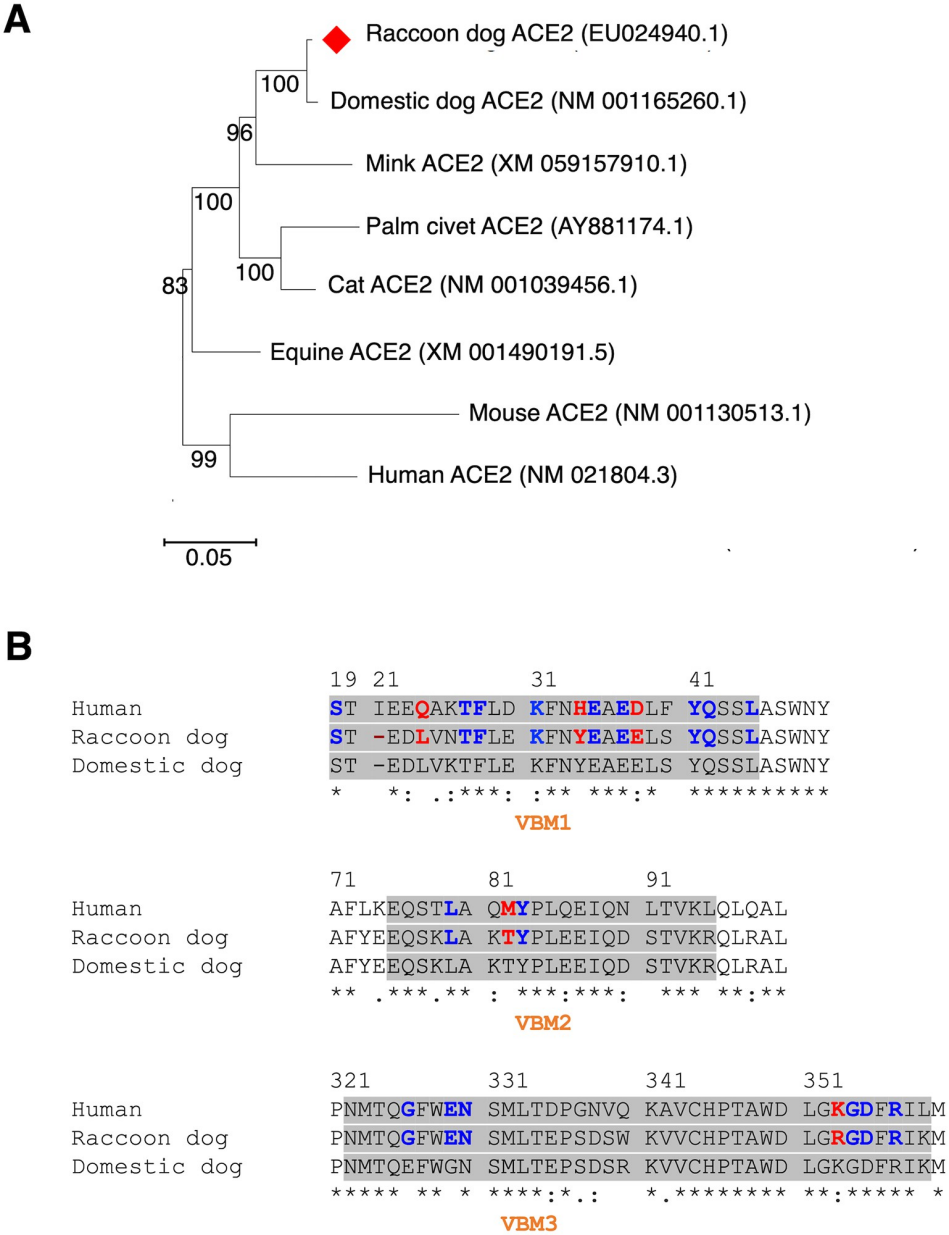
Molecular characterization of ACE2 from different species. (**A**) A phylogenetic tree based on the full-length ACE2 gene among different species was built using the maximum likelihood estimation (MLE) method. The Kimura 2-parameter (K2P) mode was selected under a discrete gamma distribution with bootstraps for 1,000 replications. (**B**) Sequence alignment of three virus-binding motifs (VBMs) among the ACE2 molecules from human, raccoon dog and domestic dog. Residues that directly contact SARS-CoV-2 RBD are labeled in blue. Among these RBD-contacting residues, those that differ between human ACE2 and raccoon dog ACE2 are labeled in red. Asterisks indicate positions that have a single, fully conserved residue. Colons indicate positions that have strongly conserved residues. Periods indicate positions that have weakly conserved residues. Hyphen indicates deletion at residue 21 in raccoon dog ACE2 and domestic dog ACE2. For the sake of clarity, the residue numbering in raccoon dog ACE2 and domestic dog ACE2 is based on the human ACE2 sequence, ignoring the deleted residue at position 21.

To examine the genetic relationships among ACE2 molecules from various host species, we constructed a phylogenetic tree based on ACE2 molecules from host species with known structural interactions with SARS-CoV-2 RBD (Fig 1A). These host species include humans, mice, domestic dogs, minks, cats, palm civets, and horses. Our analysis revealed that domestic dog ACE2 shares the closest genetic similarity with raccoon dog ACE2, as they fall within the same clade. Conversely, human ACE2 is the most distant from raccoon dog ACE2. Since ACE2 interacts with SARS-CoV-2 RBD through three virus-binding motif (VBM) regions, we conducted a detailed comparison of the VBMs found in ACE2 proteins from raccoon dogs, domestic dogs, and humans (Fig 1B). Our findings indicated that among the virus-binding residues, five of them exhibit differences between raccoon dog ACE2 and human ACE2 (specifically, residues 24, 34, 38, 82, and 353). However, only one residue differs between raccoon dog ACE2 and domestic dog ACE2, namely residue 353. In this study, we delved into the impact of these residue differences on the functional activities of ACE2 proteins serving as receptors for SARS-CoV-2.

**Fig 2:**
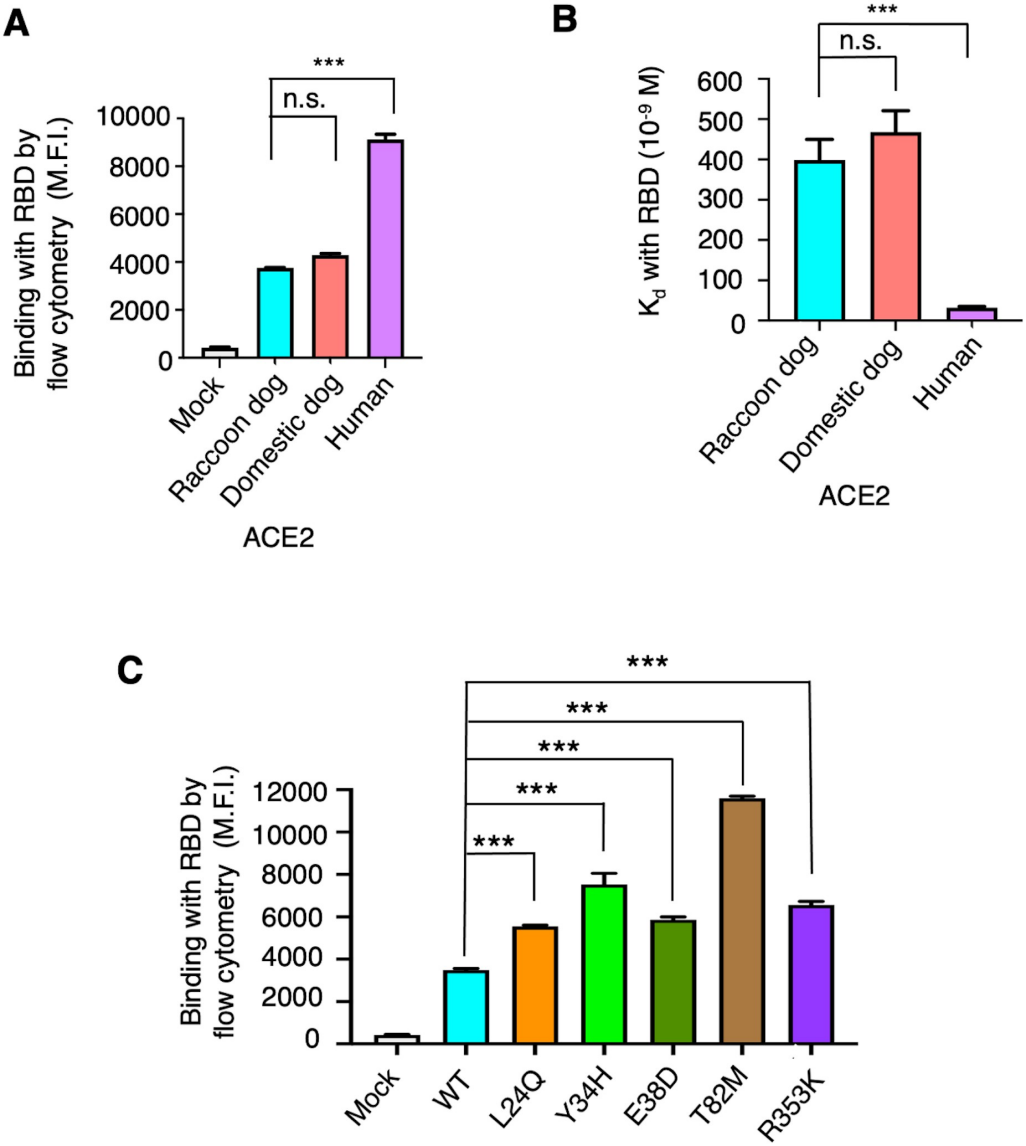
Binding interactions between SARS-CoV-2 RBD and the ACE2 molecules of different species. (**A**) Flow cytometry between recombinant SARS-CoV-2 RBD and cell surface-expressed ACE2 molecules from human, raccoon dog and domestic dog. The mean fluorescence intensity (M.F.I.) is determined using PE anti-His tag antibodies, which target the His-tagged RBD protein bound to ACE2-positive cells. The mock group represents cells transfected with an empty vector. The data are presented as mean ± SEM (n = 4) (S1A Fig). A Student’s two-tailed *t*-test was performed to analyze the statistical difference between the indicated groups; the results are labeled on top of each bar. ***p < 0.001. n.s.: not significant. (**B**) Surface plasmon resonance (SPR) between recombinant SARS-CoV-2 RBD and recombinant ACE2 molecules from human, raccoon dog and domestic dog. The data are presented as mean ± SEM (n = 3) (S1 Table; S2 Fig). A Student’s two-tailed *t*-test was performed to analyze the statistical difference between the indicated groups; the results are labeled on top of each bar. ***p < 0.001. n.s.: not significant. (**C**) Flow cytometry between recombinant SARS-CoV-2 RBD and raccoon dog ACE2 (wild type or containing one of the indicated mutations). The data are presented as mean ± SEM (n = 3) (S1B Fig). A Student’s two-tailed *t*-test was performed to analyze the statistical difference between the indicated groups; the results are labeled on top of each bar. ***p < 0.001.

To assess how effectively raccoon dog ACE2 can serve as a receptor for SARS-CoV-2, we investigated its interaction with SARS-CoV-2 spike. For comparison, we used domestic dog ACE2 and human ACE2, which are the closest and most distant relatives to raccoon dog ACE2, respectively, as seen above (Fig 1A). It is important to note that both domestic dog ACE2 and human ACE2 have already been confirmed to function as effective receptors for SARS-CoV-2 [11,19,26]. Here we used flow cytometry to evaluate the binding interactions between recombinant SARS-CoV-2 RBD and cell surface-anchored raccoon dog ACE2 (Figs 2A and S1A). The result showed that raccoon dog ACE2 and domestic dog ACE2 bind to SARS-CoV-2 RBD with similar fluorescence levels. However, both do so more weakly than human ACE2. Moreover, we used surface plasmon resonance assay (SPR) to measure the binding affinity between recombinant SARS-CoV-2 RBD and recombinant raccoon dog ACE2 (Figs 2B and S2; S1 Table). The result confirmed that raccoon dog ACE2 and domestic dog ACE2 bind to SARS-CoV-2 RBD with similar affinity, with Kds of 399 nM and 468 nM, respectively. Human ACE2 binds to SARS-CoV-2 RBD with significantly higher affinity, with a Kd of 31.7 nM. Additionally, a pseudovirus entry assay was already conducted by another study for evaluation of the efficiency of raccoon dog ACE2 in mediating SARS-CoV-2 spike-mediated viral entry [26]. The result showed that raccoon dog ACE2 and domestic dog ACE2 mediate SARS-CoV-2 pseudovirus entry with similar efficiency. However, both do so less efficiently than human ACE2. Overall, these data consistently demonstrate that raccoon dog ACE2 and domestic dog ACE2 are both reasonable receptors for SARS-CoV-2, but they are not as effective as human ACE2.

Next we examined the differences between human ACE2 and raccoon dog ACE2 that account for their varying effectiveness as SARS-CoV-2 receptors. To this end, we investigated the five RBD-contacting residues that differ between the ACE2 molecules: L24Q, Y34H, E38D, T82M, and R353K (the former and latter amino acids refer to those in raccoon dog ACE2 and human ACE2, respectively) (Fig 1B). Using human ACE2 as the reference, we introduced each of the five mutations (L24Q, Y34H, E38D, T82M, R353K) into raccoon dog ACE2. Subsequently, we evaluated the binding interactions between SARS-CoV-2 RBD and each of the mutant raccoon dog ACE2 molecules using flow cytometry (Figs 2C and S1B). The result showed that each of the five mutations enhanced the binding of SARS-CoV-2 RBD to raccoon dog ACE2. Out of the five mutations, Y34H and T82M caused the greatest increase in the binding fluorescence signals between SARS-CoV-2 RBD and raccoon dog ACE2. These data indicate that the five residue differences between human ACE2 and raccoon dog ACE2 at least partly account for the more efficient usage of human ACE2 by SARS-CoV-2 as its receptor.

**Fig 3:**
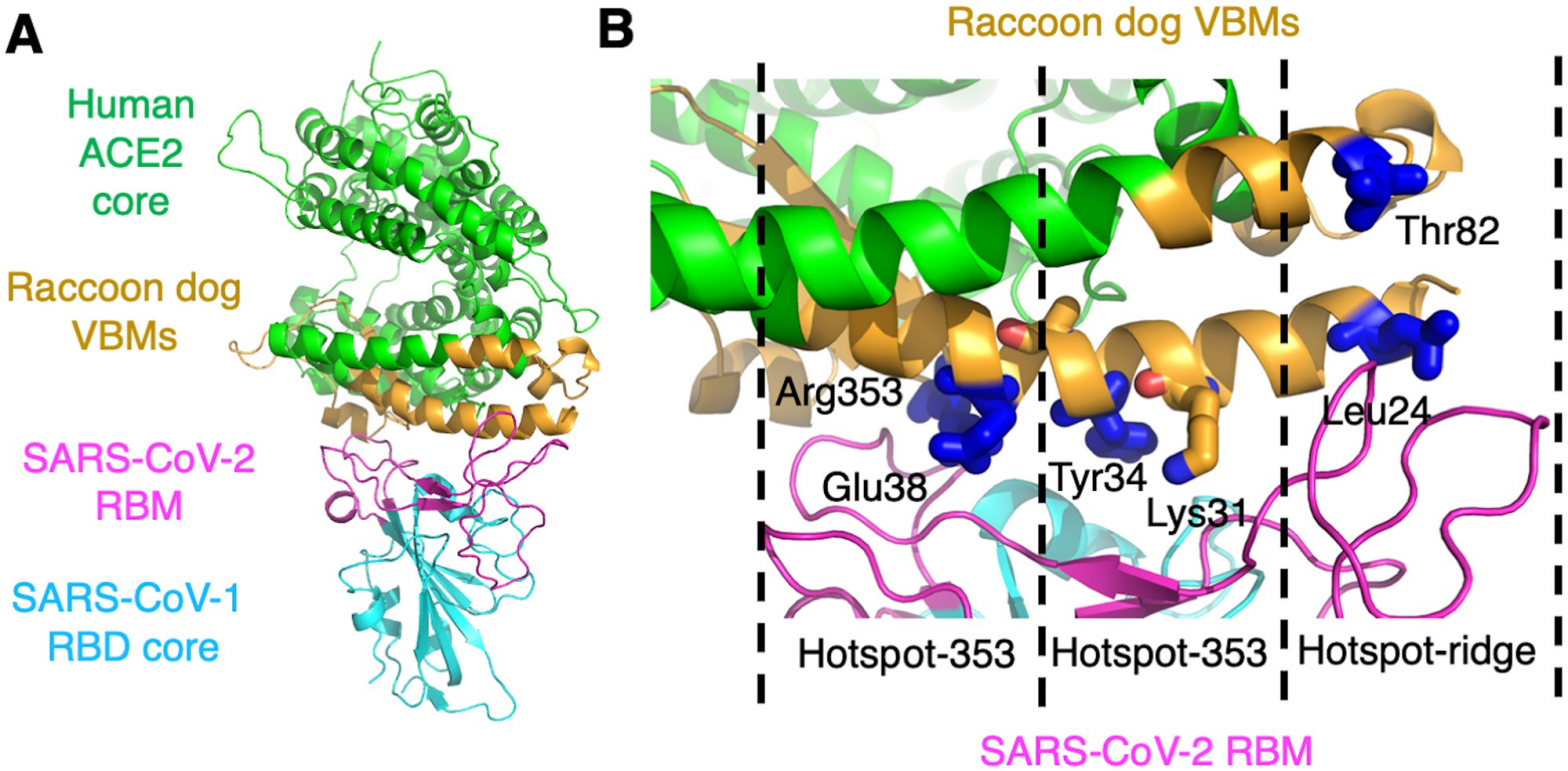
Crystal structure of chimeric SARS-CoV-2 RBD complexed with chimeric raccoon dog ACE2. (**A**) Overall structure of the complex. The chimeric RBD contains the core structure (in cyan) from SARS-CoV-1 RBD and receptor-binding motif (RBM) (in magenta) from SARS-CoV-2 RBD. The chimeric raccoon dog ACE2 contains the core structure (in green) from human ACE2 and three virus-binding motifs (VBMs) (in orange) from raccoon dog ACE2. (**B**) Structural interface between SARS-CoV-2 RBM and raccoon dog VBMs. Three virus-binding hotspots are highlighted. The key residues that differ between human ACE2 and raccoon dog ACE2 are shown in sticks.

Subsequently, we explored the structural basis for the varying effectiveness of human ACE2 and raccoon dog ACE2 as SARS-CoV-2 receptors. To this end, we determined the structural interface between SARS-CoV-2 RBD and raccoon dog ACE2 (Figs 3A and S3; S2 Table). In line with our previous research [11,14–17], we used a versatile structural platform that combines a chimeric SARS-CoV-2 RBD (containing the core structure from SARS-CoV-1 and RBM from SARS-CoV-2) with a chimeric raccoon dog ACE2 (containing the core structure from human ACE2 and VBMs from raccoon dog ACE2) (Fig 3A). The complex of these two chimeric proteins crystallizes in the same way as the complex of SARS-CoV-1 RBD and human ACE2, yet it reveals the specific structural interactions between SARS-CoV-2 RBD and raccoon dog ACE2. Even though local conformations of residues in the cores of the RBDs and ACE2 molecules may indirectly influence the conformations of residues in the RBM and VBMs, respectively, the reliability and accuracy of our chimeric protein structural platform have been confirmed by detailed comparisons with the wild type protein structure platform [11]. As we have shown in previous research, the binding interactions between the RBDs (from SARS-CoV-2, SARS-CoV-1 or related coronaviruses) and human ACE2 are largely determined by three virus-binding hotspots at the RBD/ACE2 interface: “hotspot-31” centers around Lys31 in human ACE2, “hotspot-353” centers around Lys353 in human ACE2, and “hotspot-ridge” centers around a receptor-binding ridge in the SARS-CoV-2 RBM [11,14–17]. The five residue differences between human ACE2 and raccoon dog ACE2 are all situated close to these three hotspots (Fig 3B), affecting the structures of the hotspots and consequently the binding interactions between the RBD and ACE2.

**Fig 4:**
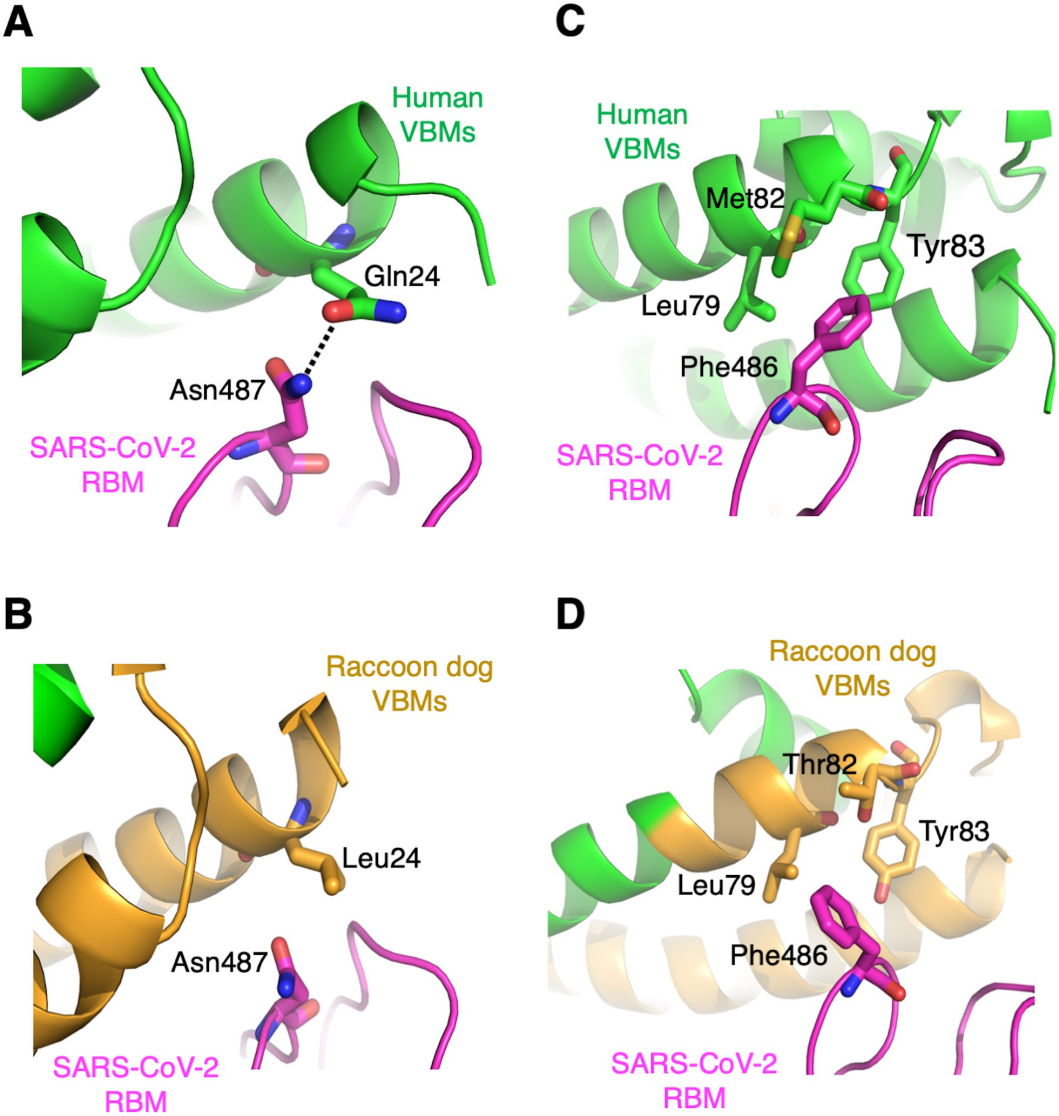
Structural details at hotspot-ridge. **(A) (C)** Structural interface between SARS-CoV-2 RBM and human VBMs at the hotspot-ridge. PDB ID: 6VW1. **(B) (D)** Structural interface between SARS-CoV-2 RBM and raccoon dog VBMs at hotspot-ridge. The dotted line indicates a hydrogen bond.

Two of the key residue differences between human ACE2 and raccoon dog ACE2, L24Q and T82M, are located at the hotspot-ridge. As we reported previously, one of the main reasons why SARS-CoV-2 RBD binds to human ACE2 more strongly than SARS-CoV-1 RBD does is because of its more compact receptor-binding ridge and hence more extensive interactions with the N-terminal helix of human ACE2 [11]. Among these interactions, Asn487^RBM^ forms a hydrogen bond with Gln24^VBM^ from human ACE2 (Fig 4A), but not with Leu24^VBM^ from raccoon dog ACE2 (Fig 4B). The L24Q mutation introduced to raccoon dog ACE2 enhanced the binding of SARS-CoV-2 RBD likely by restoring the favorable hydrogen bond with Asn487^RBM^ (Fig 2C). Moreover, as we reported previously, a critical interaction between SARS-CoV-2 RBD and human ACE2 at the hotspot-ridge is the insertion of Phe486^RBM^ into a hydrophobic pocket formed by three residues from human ACE2: Leu79^VBM^, Met82^VBM^, and Tyr83^VBM^ (Fig 4C) [11]. Thr82^VBM^ from raccoon dog ACE2, however, weakens this hydrophobic interaction network at the hotspot-ridge (Fig 4D), reducing ACE2’s binding affinity with SARS-CoV-2 RBD. The T82M mutation introduced to raccoon dog ACE2 enhanced the binding of SARS-CoV-2 RBD likely by restoring the strong hydrophobic network with Phe486^RBM^ (Fig 2C). Together, the two residue changes at the hotspot-ridge partially account for the differential activities of human ACE2 and raccoon dog ACE2 as SARS-CoV-2 receptors.

**Fig 5:**
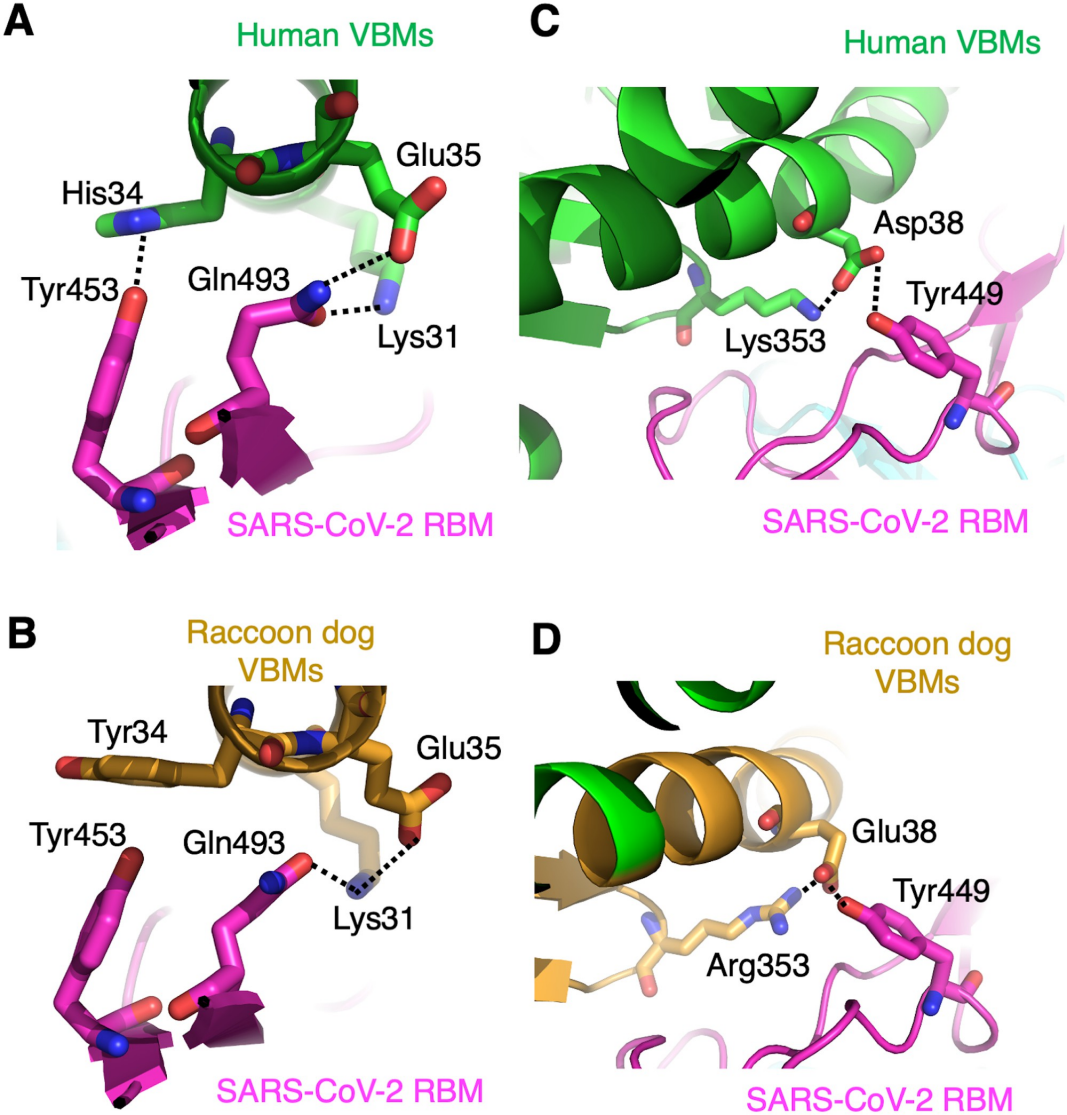
Structural details at hotspot-31 and hotspot-353. **(A)** Structural interface between SARS-CoV-2 RBM and human VBMs at the hotspot-31. PDB ID: 6VW1. **(B)** Structural interface between SARS-CoV-2 RBM and raccoon dog VBMs at the hotspot-31. **(C)** Structural interface between SARS-CoV-2 RBM and human VBMs at the hotspot-353. PDB ID: 6VW1. **(D)** Structural interface between SARS-CoV-2 RBM and raccoon dog VBMs at the hotspot-31. The dotted lines indicate hydrogen bonds or salt bridges.

The other three key residue differences between human ACE2 and raccoon dog ACE2, Y34H, E38D and K353R, are located at the hotspot-31 (Y34H) and hotspot-353 (E38D and K353R), respectively. At the hotspot-31, Lys31^VBM^ and Glu35^VBM^ from human ACE2 each form a hydrogen bond with Gln493^RBM^ (Fig 5A). As we reported previously, Gln493^RBM^ has been undergoing frequent evolutionary changes, showcasing its importance in stabilizing the hotspot-31 structure [15–17]. As an extension of the hotpot-31 structure, the side chain of Tyr453^RBM^ stacks with that of Gln493^RBM^, while forming a hydrogen bond with His34^VBM^ from human ACE2 (Fig 5A). In raccoon dog ACE2, Tyr34^VBM^ forms steric interference with Tyr453^RBM^, destabilizing the hotspot-31 structure (Fig 5B). The Y34H mutation introduced to raccoon dog ACE2 likely removed this steric interference with Tyr453^RBM^ and restored the favorable hydrogen bond with Tyr453^RBM^, stabilizing the hotspot-31 structure and enhancing the ACE2’s binding affinity for the RBD (Fig 2C). At the hotspot-353, Lys353^VBM^ from human ACE2 is buried in a hydrophobic tunnel and at the end of the tunnel it forms a critical salt bridge with Asp38^VBM^ from human ACE2; the latter is further stabilized by a hydrogen bond network involving Tyr449^RBM^ (Fig 5C). In raccoon dog ACE2, Arg353^VBM^ and Glu38^VBM^ have longer side chains than Lys353^VBM^ and Asp38^VBM^ from human ACE2, respectively. Hence they form a very strong salt bridge, which forces the side chain of Glu38^VBM^ to take a twisted conformation (Fig 5D). Either the R353K or E38D mutation introduced to raccoon dog ACE2 likely relieved the tension on the side chain of Glu35^VBM^, improving the hotspot-353 structure and enhancing the ACE2’s binding affinity for the RBD (Fig 2C). Overall, the three residue changes at the hotspot-31 and hotspot-353 also partially account for the differential activities of human ACE2 and raccoon dog ACE2 as SARS-CoV-2 receptors.

The detailed structural understanding of raccoon dog ACE2 as a SARS-CoV-2 receptor can be extended to the ACE2 molecules from other species (Table 1). At residue 24, the ACE2 molecules from domestic dogs, cats, minks, palm civets and horses all contain a leucine, which is unfavorable for SARS-CoV-2 binding. Mouse ACE2 contains an asparagine at this position; with a polar but shorter side chain, Asn24^VBM^ may be less favorable than Gln24^VBM^, but may be more favorable than Leu24^VBM^, for SARS-CoV-2 binding. At residue 34, cat ACE2 has the same histidine as human ACE2, which is favorable for SARS-CoV-2 binding, whereas the ACE2 molecules from domestic dogs, minks and palm civets all have the same tyrosine as raccoon dog ACE2, which is unfavorable for SARS-CoV-2 binding. The ACE2 molecules from horses and mice both contain a serine and an asparagine, respectively, at this position, whose interactions with SARS-CoV-2 RBD need to be characterized further. At residues 38 and 353, the ACE2 molecules from domestic dogs, cats, minks, palm civets and horses all contain Glu38^VBM^ and Lys353^VBM^, which are favorable for SARS-CoV-2 binding. In contrast, mouse ACE2 contains Asp38^VBM^ and His353^VBM^, which are unfavorable for SARS-CoV-2 binding. At residue 82, the ACE2 molecules from domestic dogs, cats, minks, palm civets and horses all contain a threonine, which is unfavorable for SARS-CoV-2 binding. Mouse ACE2 contains Ser82^VBM^, which should also be unfavorable for SARS-CoV-2 binding. Overall, these analyses lay out key residue differences among the ACE2 molecules from different species that impact their functions as SARS-CoV-2 receptors. Please note that our study was focused on the prototypic strain of SARS-CoV-2. Other studies, however, have examined the structural and biochemical interactions of later SARS-CoV-2 strains with the ACE2 receptors across different animal species [20,23,27].

**Table 1:** Selected virus-contacting residues in ACE2 from different mammalian species.

Name	Accession No.	24	34	38 & 353	82
Human	NP_001358344.1	Q	H	D & K	M
Raccoon dog	EU024940.1	L	Y	E & R	T
Domestic dog	NP_001158732.1	L	Y	E & K	T
Cat	XP_023104564.1	L	H	E & K	T
Mink	XP_059013893.1	L	Y	E & K	T
Palm civet	AAX63775.1	L	Y	E & K	T
Horse	XP_001490241.1	L	S	E & K	T
Mouse	NP_001123985.1	N	Q	D & H	S

## Discussion

Raccoon dogs have been implicated in the transmission of SARS-CoV-1 to humans through animal markets and dining venues [4]. Now, they are suspected of aiding the spread of SARS-CoV-2, with their DNA found alongside the virus at the Wuhan seafood market, the identified epicenter of the COVID-19 outbreak [1–3]. Since receptor binding is a key factor in determining host ranges for coronaviruses, our study has investigated the raccoon dog ACE2 as a receptor for prototypic SARS-CoV-2. We have compared it with domestic dog ACE2 and human ACE2, the closest and most distantly related to raccoon dogs respectively, among those with known structural interactions with SARS-CoV-2 RBD. Our research shows that raccoon dog ACE2 can effectively bind SARS-CoV-2 RBD, similar to domestic dog ACE2, but not as strongly as human ACE2. Our study provides an in-depth analysis of raccoon dog ACE2’s interaction with SARS-CoV-2 spike protein, suggesting that raccoon dogs might be capable carriers of prototypic SARS-CoV-2 and hence might have contributed to the initial human transmission.

Coronaviruses are known for their ability to cross species [5,6,28]. Typically, when a coronavirus persistently infects a particular host species, its spike protein mutates to better fit the receptor in that host. If the virus then jumps to a new host species, its spike must adapt again to the new species’ receptor. Thus, the differences in key spike-binding residues on receptors across various species act as a barrier to the virus’s capacity to infect cells from each of those species [5–7]. The species-specific transmission barriers for SARS-CoV-2 have been under-studied until now. Our study has tackled this by comparing raccoon dog ACE2 and human ACE2. We have identified five key residues (residues 24, 34, 38, 82, 353) that differ between the two ACE2 receptors. Through structural and biochemical methods, we have discovered that the human ACE2 residues (Asn24, His34, Met82, Asp38/Lys353) bind more favorably with SARS-CoV-2 than those in raccoon dog ACE2 (Leu24, Tyr34, Thr82, Glu38/Arg353). This is because the human ACE2 residues better stabilize the three virus-binding hotspots at the RBD/ACE2 interface than those in raccoon dog ACE2. These differences in key RBD-binding residues between raccoon dog ACE2 and human ACE2 may pose barriers affecting the transmission of SARS-CoV-2 between raccoon dogs and humans. These barriers would need to be investigated further if the structural features of a SARS-CoV-2 spike protein that had adapted to recognize the raccoon dog ACE2 receptor were identified. Nevertheless, based on the structural information currently available, our research provides an in-depth investigation of these species-specific transmission barriers for prototypic SARS-CoV-2.

Prior studies have looked at the interaction of ACE2 molecules from several individual species with SARS-CoV-2 RBD, but a detailed comparative structural analysis among ACE2 molecules from different species has not been conducted. This study bridges that gap with a focused structural view of raccoon dog ACE2 and human ACE2 as SARS-CoV-2 receptors. We expanded our analysis to include ACE2 from other species, including domestic dogs, cats, minks, palm civets, horses, and mice, concentrating on the aforementioned five key RBD-contacting residues. In human ACE2, the residues at positions 24, 34, and 82 as well as the combination of residues 38 and 353 are all favorable for SARS-CoV-2 binding. In contrast, mouse ACE2 has residues at positions 24, 82, and 353 that are less conducive to SARS-CoV-2 binding, with His353 being very unfavorable for SARS-CoV-2 binding [17]. Accordingly, among the species examined, human ACE2 is the most potent receptor for SARS-CoV-2. On the other hand, mouse ACE2 demonstrates the least effectiveness, corroborating prior studies that found mouse ACE2 to be an inefficient receptor for SARS-CoV-2 entry [17]. The ACE2 receptors from other species rank between those of humans and mice, in agreement with previous findings that these receptors are adequately competent for SARS-CoV-2 binding [18,23]. We wish to highlight that our analyses depend on an in-depth comparative structural analysis of raccoon dog ACE2 and human ACE2, coupled with sequence alignment of ACE2 molecules from different species. The implications of these analyses, particularly concerning the effects of mutations on the role of various ACE2 molecules as SARS-CoV-2 receptors, need to be confirmed with experimental data. Additionally, although this study focused on particular ACE2 molecules, the insights could extend to any other species’ ACE2 as potential SARS-CoV-2 receptors. Overall, our study enriches the understanding of ACE2 variants across species, their roles as SARS-CoV-2 receptors, and the interspecies barriers to the virus’s transmission.

## Materials and methods

### Cell lines and plasmids

HEK293T cells (American Type Culture Collection (ATCC)) were grown in Dulbecco’s modified Eagle medium (DMEM) (containing 10% fetal bovine serum, 2 mM L-glutamine, 100 units/mL penicillin, and 100 μg/mL streptomycin). 293F cells (ThermoFisher) were grown in FreeStyle 293 Expression Medium (ThermoFisher). Both cells were authenticated by ATCC using STR profiling and were also tested for mycoplasma contamination. No commonly misidentified cell lines were used.

The genes encoding ACE2 molecules from human, raccoon dog and domestic dog (GenBank accession numbers: NM_021804, EU024940.1 and NP_001158732.1, respectively) and SARS-CoV-2 spike (prototypic strain; GenBank accession number: QHD43416.1) were synthesized (GenScript).

For protein expression, the gene encoding SARS-CoV-2 RBD (residues 319–529) was subcloned into pCAGGS vector (GenScript) with a C-terminal His tag. The genes encoding the ectodomains of human ACE2 (residues 1–615), raccoon dog ACE2 (residues 1–614) and domestic dog ACE2 (residues 1–614) were subcloned into pLenti-transfer vector (Addgene) with a human IgG Fc tag [11,16,17]. The chimeric SARS-CoV-2 RBD and the chimeric raccoon dog ACE2 were subcloned into pFastBac I vector (Life Technologies) with an N-terminal honeybee melittin signal peptide and a C-terminal His tag [16,17].

For flow cytometry, all genes encoding the wildtype or mutated ACE2 were subcloned into the pcDNA3.1(+) vector (GenScript) with a C-terminal C9-tag.

### Phylogenetic analysis

Multiple alignment of the ACE2 genes from different animal species was constructed using ClustalW in the Molecular Evolutionary Genetics Analysis (MEGA) version 11 software; a phylogenetic tree was built using the maximum likelihood estimation (MLE) method in the Kimura 2-parameter (K2P) mode under a discrete gamma distribution with bootstraps for 1,000 replications [29].

### Protein expression and purification

For biochemical assays, plasmids encoding Fc-tagged ACE2 ectodomains (from human, raccoon dog and domestic dog) or His-tagged SARS-CoV-2 RBD were transfected into the 293F cells using lipofectamine3000 reagent (Invitrogen) [16,17]. After 3 days, the supernatants were collected and the proteins were purified using a NiNTA column (for the His-tagged protein) or protein A column (for the Fc-tagged proteins), and purified further on a Superdex 200 Increase 10/300 gel filtration column (Cytiva).

For crystallographic studies, His-tagged chimeric SARS-CoV-2 RBD and His-tagged chimeric raccoon dog ACE2 ectodomain were expressed in sf9 insect cells using the Bac-to-Bac system (Life Technologies) [16,17]. The proteins were harvested from the supernatant, purified using a NiTNA column and a Superdex 200 Increase 10/300 gel filtration column (Cytiva).

For the NiNTA column, the binding buffer consisted of 20 mM Tris-HCl and 500 mM NaCl at pH 7.4. The elution buffer had the same composition as the binding buffer but was supplemented with 500 mM imidazole. Regarding the protein A column, Dulbecco’s Phosphate-Buffered Saline (DPBS) (Gibco) was used for binding, while the IgG elution buffer (ThermoFisher) was used for elution.

### Flow cytometry

Flow cytometry was conducted as previously described [29,31]. Briefly, each of the ACE2-encoding plasmids was transfected into HEK293T cells at a confluency of 80% in 6-well plates using lipofectamine3000 reagent (Invitrogen). After 48 hours, the cells were detached using Accutase (Millipore), incubated with Human TruStain FcX (BioLegend) for 10 minutes on ice, and dissolved in the flow cytometry buffer containing 5% fetal bovine serum (Invitrogen) and 1mM EDTA (VWR) at numbers of approximately 10^6^ cells. To measure the level of SARS-CoV-2 RBD attaching to the cell surface, ACE2-transfected HEK293T cells were first incubated with 2 μg of purified His-tagged RBD on ice for 1 hour, stained with rhodopsin antibody Alexa Fluor 647 (Santa Cruz Biotechnology) at 1:200 dilution and 5 μl PE anti-His tag antibody (BioLegend) on ice for 20 minutes, and finally resuspended in the flow cytometry buffer. Fluorescent signals on the cellular surface were immediately analyzed using Symphony A3 (BD Biosciences), FlowJo software (Ashland) and GraphPad Prism version 9.5.0 with one-way analysis of variance (ANOVA). All measurements were independently repeated at least for biological triplicates.

### Surface plasmon resonance (SPR)

The binding affinity between recombinant His-tagged RBD and Fc-tagged ACE2 was measured by SPR using the Biacore S200 system (Cytiva) as previously described [16,17]. Each ACE2 protein (in the amount of 4.85 μg) was immobilized onto the Sensor Chip Protein A (Cytiva). Then, the RBD protein at concentrations of 40, 80, 160, 320 and 640 nM was injected over the sensor surface, bound to immobilized ACE2 protein and washed off the surface. The running buffer used for all proteins in the SPR assays consisted of 10 mM HEPES, 150 mM NaCl, 3 mM EDTA, and 0.05% Tween 20, adjusted to pH 7.4. The apparent equilibrium dissociation constant (Kd) was measured using the Biacore analytical software (Cytiva). For this, a one-to-one Langmuir binding model was used for Fc-tagged proteins that were immobilized, with monomeric proteins flown over them. All measurements were independently repeated for biological triplicates.

### Crystallization and structure determination

Crystallization and structure determination were performed as previously described [16,17]. Briefly, the crystals of chimeric SARS-CoV-2 RBD complexed with chimeric raccoon dog ACE2 were grown at room temperature over wells containing 100 mM Tris (pH 8–8.5), 18–22% PEG 6000, 100 mM NaCl and ethylene glycol (0.5–2%). X-ray diffraction data were collected on beamline 17-ID-1 at the National Synchrotron Light Source II (NSLS2), Brookhaven National Laboratory. Data were processed using HKL2000 [32]. The structure was determined by molecular replacement using the structure of chimeric RBD complexed with human ACE2 as the search model (PDB ID 6VW1). Molecular replacement and model refinement were performed using PHENIX and CCP4 [33,34]. Model building was carried out in COOT [35]. PYMOL (The PyMOL Molecular Graphics System, Version 2.0 Schrödinger, LLC.) was used for making structural figures. Structure data and refinement statistics are shown in S2 Table.

## Supporting information

S1 Fig Flow cytometry assay on the interactions between recombinant SARS-CoV-2 RBD and cell-surface-anchored ACE2. **(A)** HEK293T cells expressing full-length C9-tagged ACE2 (from human, raccoon dog or domestic dog) were incubated with recombinant His-tagged SARS-CoV-2 RBD. **(B)** HEK293T cells expressing full-length C9-tagged human ACE2 (wild type or containing one of the indicated mutations) were incubated with recombinant His-tagged SARS-CoV-2 RBD. A fluorescence-labelled anti-His-tag antibody and a fluorescence-labelled anti-C9-tag antibody were used to label cell-bound RBD and cell-surface-expressed ACE2, respectively. The percentages of labelled cells that were positive for both RBD and ACE2 or negative for both RBD and ACE2 are shown. The (-) control represents cells transfected with the vector only.
(TIF)

S2 Fig Measurement of the binding affinities between SARS-CoV-2 RBD and ACE2 molecules from human, raccoon dog and domestic dog using surface plasmon resonance (SPR). Each of the purified recombinant ACE2 proteins (with an Fc tag) was immobilized onto the Sensor Chip Protein A. Then the purified recombinant RBD protein (with a His tag) was injected and flowed by the sensor chip. The RBD was diluted to five different concentrations (from 40 to 640 nM) before being injected. The resulting data were fit to a 1:1 binding model. Each experiment was independently repeated for biological triplicates.
(TIF)

S3 Fig Unbiased composite omit map of the structure of chimeric SARS-CoV-2 RBD complexed with chimeric raccoon dog ACE2. Only the density of the interfaces between SARS-CoV-2 RBM and raccoon dog VBMs is shown. The SARS-CoV-2 RBM is in magenta, and the raccoon dog VBMs are in green. Resolution is 2.57 Å. Contour level is 1σ.
(TIF)

S1 Table Binding affinity between SARS-CoV-2 RBD and ACE2 receptors as measured by surface plasmon resonance. (PDF)

S2 Table Crystallography data collection and refinement statistics. (PDF)
