# Supplementary figures and images for: Structural basis for raccoon dog receptor recognition by SARS-CoV-2
Source: PLoS Pathog. 2024 May 6;20(5):e1012204. doi: 10.1371/journal.ppat.1012204 (PMC11098500; doi:10.1371/journal.ppat.1012204)

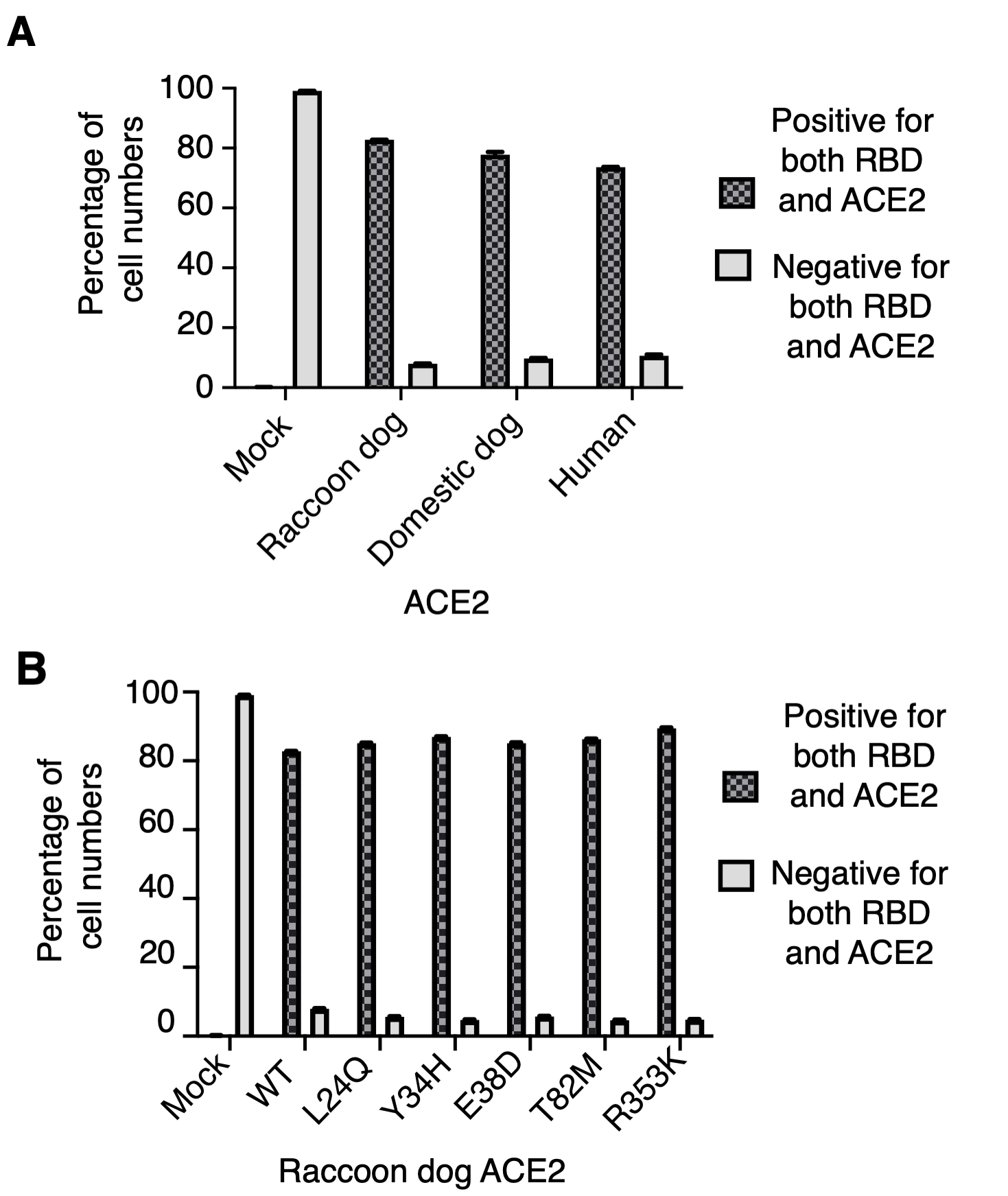

Supplement: S1 Fig — (A) HEK293T cells expressing full-length C9-tagged ACE2 (from human, raccoon dog or domestic dog) were incubated with recombinant His-tagged SARS-CoV-2 RBD. (B) HEK293T cells expressing full-length C9-tagged human ACE2 (wild type or containing one of the indicated mutations) were incubated with recombinant His-tagged SARS-CoV-2 RBD. A fluorescence-labelled anti-His-tag antibody and a fluorescence-labelled anti-C9-tag antibody were used to label cell-bound RBD and cell-surface-expressed ACE2, respectively. The percentages of labelled cells that were positive for both RBD and ACE2 or negative for both RBD and ACE2 are shown. The (-) control represents cells transfected with the vector only. (TIF) [file ppat.1012204.s001.tif]

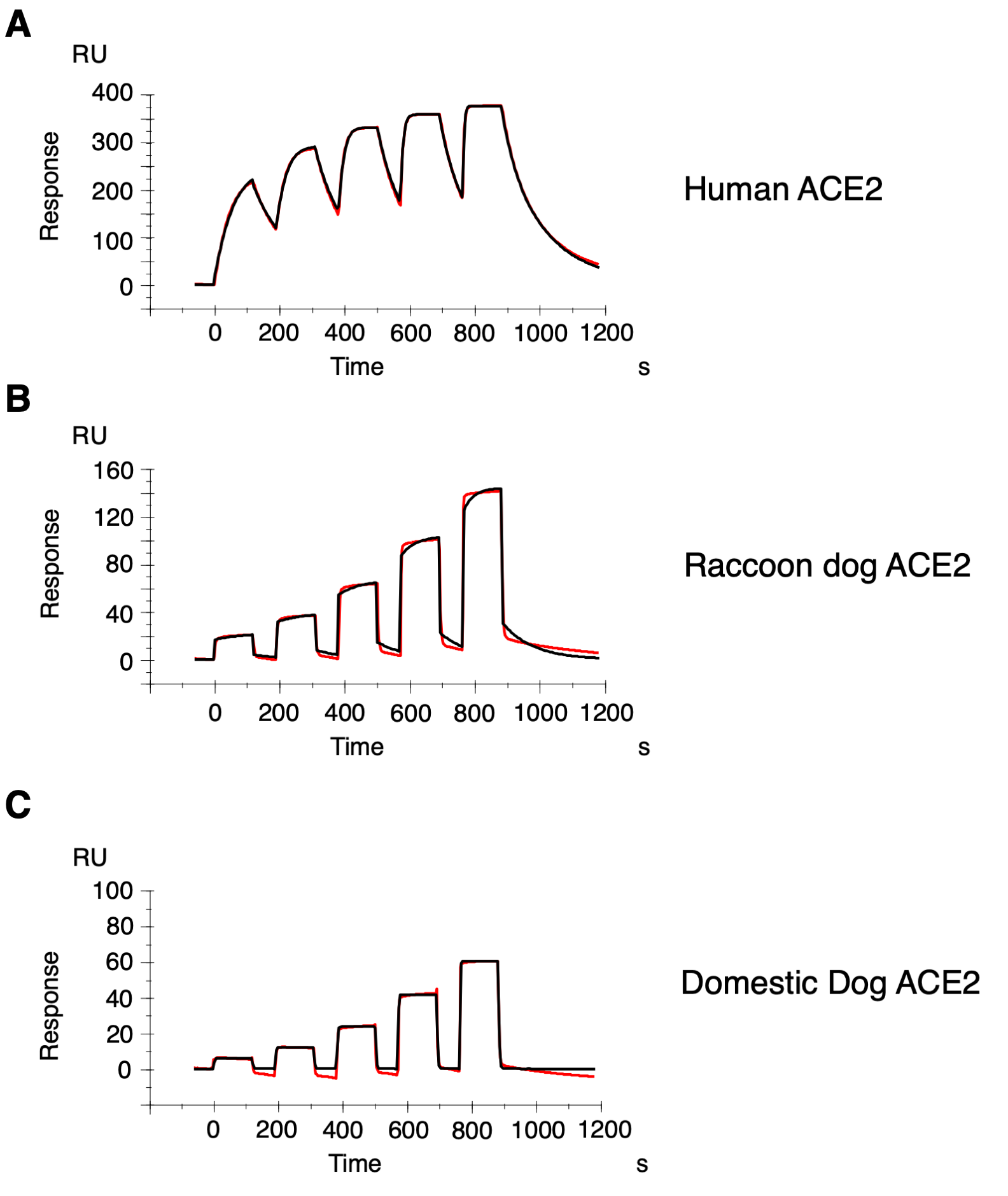

Supplement: S2 Fig — Each of the purified recombinant ACE2 proteins (with an Fc tag) was immobilized onto the Sensor Chip Protein A. Then the purified recombinant RBD protein (with a His tag) was injected and flowed by the sensor chip. The RBD was diluted to five different concentrations (from 40 to 640 nM) before being injected. The resulting data were fit to a 1:1 binding model. Each experiment was independently repeated for biological triplicates. (TIF) [file ppat.1012204.s002.tif]

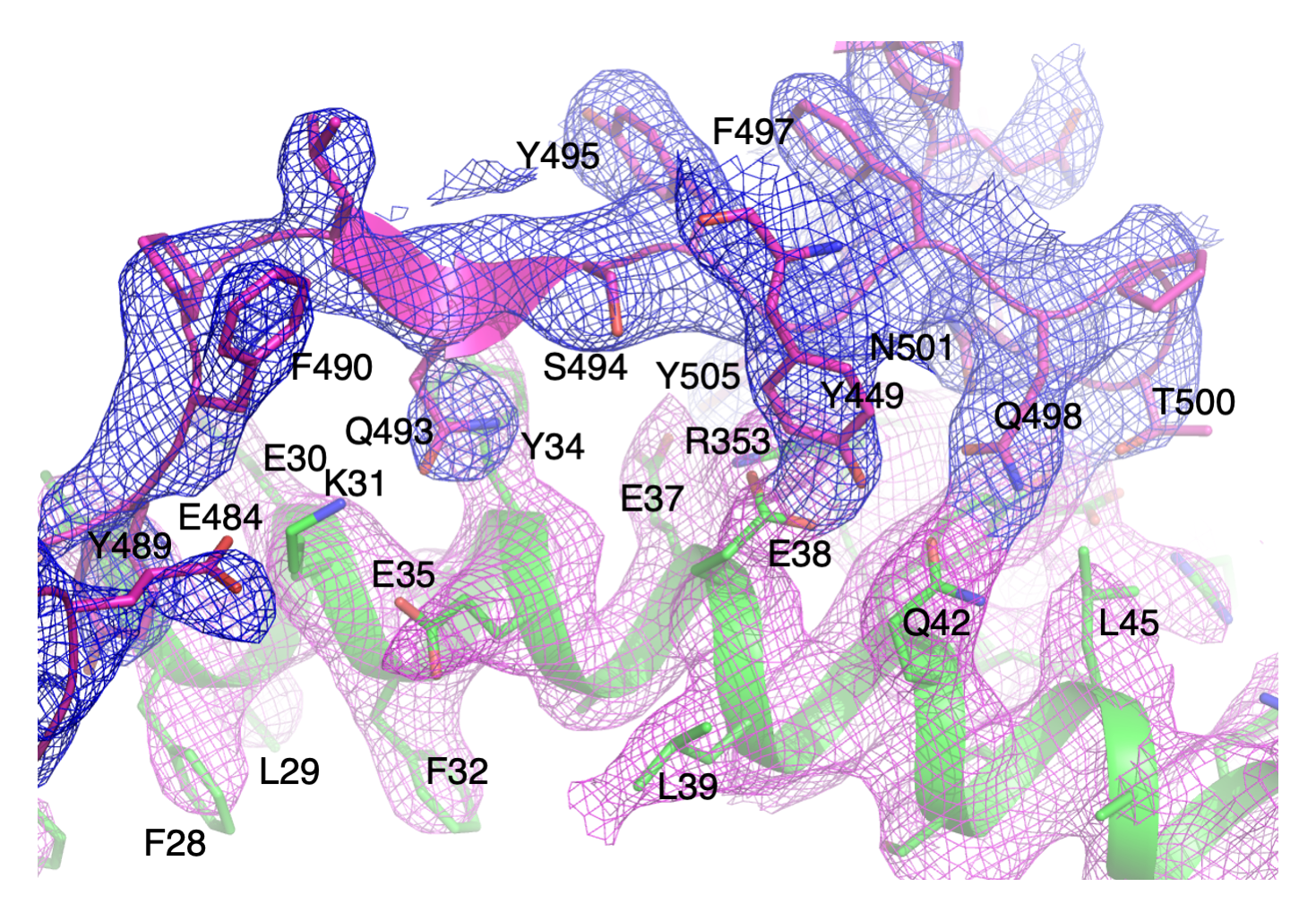

Supplement: S3 Fig — Only the density of the interfaces between SARS-CoV-2 RBM and raccoon dog VBMs is shown. The SARS-CoV-2 RBM is in magenta, and the raccoon dog VBMs are in green. Resolution is 2.57 Å. Contour level is 1σ. (TIF) [file ppat.1012204.s003.tif]
