# Supplementary material for: Structural basis for raccoon dog receptor recognition by SARS-CoV-2
Source: PLoS Pathog. 2024 May 6;20(5):e1012204. doi: 10.1371/journal.ppat.1012204 (PMC11098500; doi:10.1371/journal.ppat.1012204)
Supplement: S1 Table — (PDF) [file ppat.1012204.s004.pdf]

S1 Table. Binding affinity between SARS-CoV-2 RBD and ACE2 receptors as measured by surface plasmon resonance.

ACE2	K_{on} ($M^{-1}s^{-1}$)	k_{off} (s^{-1})	Kd (nM)	**Kd (nM)** (Mean ± SEM)
Human	4.10E+05	1.51E-02	36.9	**31.7 ±3.0**
	4.63E+05	1.23E-02	26.5	
	8.64E+05	2.74E-02	31.7	
Raccoon dog	3.58E+04	1.06E-02	297.1	**399±51**
	5.80E+05	2.61E-01	449.4	
	7.18E+05	3.23E-01	449.6	
Domestic dog	8.82E+05	4.57E-01	518.1	**468±53**
	1.04E+06	5.46E-01	524.1	
	6.52E+05	3.35E-01	361.0	
