# Supplementary material for: Structural basis for raccoon dog receptor recognition by SARS-CoV-2
Source: PLoS Pathog. 2024 May 6;20(5):e1012204. doi: 10.1371/journal.ppat.1012204 (PMC11098500; doi:10.1371/journal.ppat.1012204)
Supplement: S2 Table — (PDF) [file ppat.1012204.s005.pdf]

S2 Table. Crystallography data collection and refinement statistics.

	**RcD**
**Data collection**	
Space group	$P2_1$
Unit cell dimensions	
a, b, c (Å)	80.96, 118.11, 112.23
α, β, γ (º)	90, 92.89, 90
Resolution (Å)	112.09 - 2.57 (2.84 - 2.57)
R_{sym} or R_{merge}	0.049 (0.733)
$I / \sigma I$	9.8 (1.6)
Completeness (%)	92.8 (72.6)*
Redundancy	3.9 (4.0)
$CC_{1/2}$	0.999 (0.724)
**Refinement**	
Resolution (Å)	58.4 - 2.57 (2.66 - 2.57)
No. Reflections	42427 (277)
R_{work} / R_{free}	0.214/0.264
No. atoms	13126
Protein	12781
Ligand/ion	329
Water	16
B-factor	87.22
Protein	86.74
Ligand/ion	107.53
Water	53.25
Ramachandran plot	
Favored (%)	94.19
Allowed (%)	5.04
Outliers (%)	0.77
R.m.s. deviations	
Bond lengths (Å)	0.002
Bond angles (º)	0.51

Statistics for the highest-resolution shell are shown in parentheses.

*Ellipsoidal
